# Online trauma psychoeducation for people with depression and comorbid PTSD symptoms: A pilot randomized controlled trial

**DOI:** 10.1192/j.eurpsy.2025.1950

**Published:** 2025-08-26

**Authors:** H. W. Fung, C. T. Y. Cheung, A. K. C. Chau, C. H. O. Huang, I. Y. Y. Ho, T. Y. N. Tsui, C. Liu, G. F. Yuan

**Affiliations:** 1 School of Nursing, The Hong Kong Polytechnic University; 2 Department of Social Work, Hong Kong Baptist University, Kowloon; 3 Department of Psychiatry; 4 Department of Social Work and Social Administration, The University of Hong Kong, Pokfulam; 5 Institute of Health Equity, The Chinese University of Hong Kong, Shatin, Hong Kong; 6 School of Psychology, Australian Catholic University, Melbourne, Australia; 7 School of Education Science, Leshan Normal University, Leshan, China

## Abstract

**Introduction:**

Depression is commonly comorbid with post-traumatic stress disorder (PTSD) symptoms. There is a lack of studies evaluating trauma-informed interventions for people with depression and PTSD symptoms.

**Objectives:**

We examined whether an online, easily accessible, trauma psychoeducation program would be helpful for people with both depressive and PTSD symptoms.

**Methods:**

Participants with depression (PHQ-9 ≥ 10) and co-occurring PTSD symptoms were recruited online and randomly assigned to the intervention group (i.e., a 10-session online program based on *Be a Teammate With Yourself* ) or the control group. Outcome measures included the Brief-COPE, a subscale of the Endorsed and Anticipated Stigma Inventory, and the Post-traumatic Maladaptive Beliefs Scale. These outcomes were assessed at baseline, posttest, and 2-month follow-up. Qualitative feedback was also obtained from the participants.

**Results:**

35 participants were randomly assigned to the intervention group, and 34 to the control group. With only email reminders, 9 participants in the intervention group and 14 in the control group completed posttest and follow-up surveys. Completers-only analyses were conducted. One-way repeated measures ANOVA showed that the intervention group had significant reductions in post-traumatic maladaptive beliefs, with a large effect size (F = 4.152, p = .035, Partial Eta Squared = 0.342). The control group did not have such changes. Both groups did not have significant changes in coping and self-stigma. Of 12 participants who provided feedback, 100% agreed that the program could help them remain hopeful for recovery, and 91.6% agreed that they were satisfied with the program. The qualitative feedback also supported the usefulness and acceptability of the programme.

**Conclusions:**

Participation in this program was associated with significant decreases in post-traumatic maladaptive beliefs. Completers were satisfied with the program. Given a small sample with a high dropout rate (66.6%), the results should be interpreted with caution.

**Disclosure of Interest:**

None Declared

